# Oral Diseases

**Published:** 1886-05

**Authors:** J. N. Martin

**Affiliations:** Lecturer on Oral Diseases in the Dental Department, University of Michigan


### THE DENTAL REGISTER.

VOL. XL.] MAY, 1886. [No. 5.

### COMMUNICATIONS.

### Oral Diseases.

BY J. N. MARTIN, M.D., LECTURER ON ORAL DISEASES IN THE DENTAL DEPARTMENT, UNIVERSITY OF MICHIGAN.

Delivered before the Michigan State Dental Association at Ann Arbor, Michigan, March 17th, 1886.

The hastily prepared paper which I shall present this evening is not intended for a complete monograph on diseases of the mouth, or on any branch of them ; but intended to present only a few practical points on the subject and with especial reference to a case which I shall present in this paper.

With the exception of diseases of the teeth, there is no class of affections so generally neglected by both physicians and dentists as those of the mouth. I have, for this reason, noted a few points with reference to these diseases that I hope will be of interest to you as practical dentists. Of course I cannot do much in a brief hour in this field, when it takes sixty or more lectures to treat the subject in the way I present it to the dental students. This is a field small in area, but rich in pathological changes, that are not only of great importance locally, but in which deviations from the normal standard may produce serious constitutional disturbances. It is not so much what goeth into the mouth, or out of the mouth, as what takes place in the mouth that interests us *most*, professionally. First, any interference with the act of mastication soon overloads us with a mass of raw material that cannot be utilized for building and repairing except at a great disadvantage. If food is not properly masticated, it passes along the digestive tract, imposing more work upon the other organs and often results in deragements of a serious character which are particularly noticeable in those who are debilitated from age or or other causes. Derangements of digestion are exceedingly common, and no small number is caused by insufficient mastication due to disease in and about the organs of mastication.

Again, derangements of dentition may result in many abnormalities ranging from slight irritability or diarrhœa to convulsions, emaciation, and death. The process of dentition is a *normal physiological process*, but deviations from this standard are numerous, owing to the many modifiers at work which interfere with that process in the form of bad hygienic surroundings, *too early* administration of solid food, and many others. When the child arrives at a certain age, almost every illness is referred either directly or indirectly to dentition. The mother is perhaps feeding her babe meat, bread, potatoes, and other solid food before its organs are able to digest and assimilate them; and they act as irritants, producing derangements which, of course, in her estimation, are due to "cutting teeth," as she terms it. How common are such expressions as these, 'the baby is cross now, it is getting ready to cut teeth,' or 'is cutting teeth,' and they are *partially* true; but dentition should not be the only hiding place for physicians and dentists.

The temporary teeth are usually erupted between the sixth and thirtieth month, beginning with the central incisors; and physicians and dentists too often diagnose dentition as the cause of most derangements of that period, and far too often recommend lancing of the gums, not knowing what ought to be done. Thorough lancing of the gums is a proper procedure when indicated and at the proper time; but often it is done too early and more harm than good results; for example, if the gum over a tooth is lanced too early—before the tooth is ready to be erupted—a cicatrix forms over the tooth, and it being firmer than the original structure, there is more disturbance when the tooth is erupted than there would have been had lancing not been performed. When the gums are tense and congested, and by examination the tooth can be felt beneath, then is the time to assist nature and relieve congestion by lancing the gum. Another mistake sometimes made is in not recognizing an inflammation at the root or base of a tooth when there are no external signs of inflammation at the apex of the gums; if the inflammation is at the root of the tooth, due to backward pressure by the structures in front, then the inflammatory signs are at the base of the gum and not at the apex, and instead of the child's experiencing relief by gradual pressure forcing the blood from the tissues over the tooth, there will be an actual increase of pain.

Another great source of error is in mistaking inflammation for neuralgia, and vice versa; and in not discovering the cause or causes of neuralgia, and still more those of odontalgia; there are no less than twenty causes of odontalgia or toothache. Among them are dental caries, periostitis, caries and necrosis of maxillæ; abscesses of teeth, gums, jaws, and surrounding structures; inflammations of gums, dental pulp, and surrounding structures; death and decomposition of dental pulp; ossification of dental pulp; many different kinds of tumors of the teeth, gums, or jaws; and many cases from reflex action due to derangements of digestion and many other causes. A plug of hard wax in the ear has been known to cause the most severe form of toothache by reflex action. It is easy to see how fruitless the curative treatment would be in most of these cases without first gaining a knowledge of the cause of pain.

Again, diseases of the teeth may produce diseases of the jaws; as periostitis, caries, necrosis, and abscesses of the gums or jaws, diseases of the antrum, as abscess, dropsy, etc., diseases of the salivary glands, tonsils, cheeks, throat, nose, ear, and a long train of general disturbances, as neuralgias, blood poisoning, and derangements of digestion. Many times disease of the teeth has excited neuralgias of the most varied and peculiar form. The reflex actions in the way of producing tetanus, and trismus, or lock-jaw, are wonderful. There are many cases on record where lock-jaw has been produced by the irritation of a diseased tooth, by reflex action. Not long since, a prominent Chicago dentist reported a case where a diseased tooth, whenever irritated, caused severe tetanic contractions of different muscles in the body; there are many similar cases incorporated in oral literature. Many cases of convulsions have been traced to diseased teeth; I do not refer here to the eruptive period, but to disease of the teeth of adults. On the other hand, many diseases predispose or cause directly disease of the oral structures, as syphilis, continued and eruptive fevers, etc.; in fact, anything that alters the secretions of the mouth or alimentary canal generally produces a morbid state of the secretions that acts injuriously upon the dental tissues.

In this locality, there are certainly many changes from the normal; there are many abnormalities and diseases of the gums, as tumid gums, hypertrophied and atrophied gums; alveo-dental abscesses, abscesses of the antrum and other structures; inflammations of all the parts; tumors of all the parts, as polypoid, fungoid, fibroid, sarcoma, and cancers; deformities of the teeth, gums, jaws, and palate; diseases and deformities of the tongue, lips, and throat by the score; and many other diseases too numerous to mention in this short paper. All of these diseases, with their many complications, make this field of study not only an exceedingly interesting one, but a very important one, from its local importance and also from its bearing on the general economy. With this long line of diseases, the numerous pathological changes and the exceedingly complex anatomy of the mouth; who can reasonably say that the dentist has not a very interesting and important field of study? Without further remarks in a general way, I wish to call your attention to a case of considable interest; a case of necrosis of the lower jaw with abscess near angle.

The patient, Louis Hagberg, age 23, was born in Sweden, where he lived until 1883, when he came to America, arriving here in that year in May; he has lived in and about Muskegon, Mich., since his arrival, working in the saw mills in the summer, and in the lumber camps in the winter. The patient's father and mother are both living and well. The patient was never ill before coming to this country, and since his arrival has been well until last July (1885), with the exception of a slight attack of malarial fever the first year in America, which continued two weeks, but was not severe. He is of average size and light complexion, and, as you see him at present, is well developed and rugged, much changed from the appearance he presented when he applied for treatment.

The history of the recent trouble is as follows: The 10th of July last (1885), after eating dinner, the patient complained of pain in the region of the right molars of the lower maxilla, and supposed he had toothache, as he termed it; the pain continued during the afternoon, and at night the right side of the face began to swell more over the lower maxilla; pain and swelling increased for three days, when the face was greatly swollen and distorted, the teeth of the lower jaw began to loosen, and at the end of a week, three of the teeth had dropped out, two on the left side (the lateral incisor and first bicuspid) and one on the right side (the first bicuspid) and the rest of the teeth of the lower maxilla were so loose that by gravity alone they would tip inward or outward, or from side to side, according to his position, for the disease at this time had extended to the left side. At the expiration of three days, pain was very severe and swelling so great that he could not open his mouth. He then consulted a physician who advised the use of a poultice locally and gave him some general remedy; the poultice was applied on the right side and a carious molar extracted from that side; the swelling was soon reduced. Two days later, the swelling was nearly gone from the right side, but the left side was greatly swollen; the swelling having extended around the jaw to that side; the poultice was applied to the left side, but did not accomplish the desired end—the swelling was not reduced. During this time the gums were much swollen, spongy, and very sensitive. The pain was almost unbearable for three weeks, the patient suffered so much that during these three weeks he could not sleep except for a few minutes at a time; he was somewhat delirious at times, and became so reduced at the end of the third week from loss of sleep and severe pain that he could not walk, and it was with difficulty that he could stand alone. During this time he was obliged to live on liquid food, as he could not open his mouth. Suppuration having been well established at the end of the third week and many abscesses formed, an opening occurred on the right side near the back molar and considerable pus escaped; several other openings soon occurred in different parts of the jaw and pus flowed freely from all of them with an exceedingly offensive odor. As soon as the pus was evacuated all pain ceased, but profuse suppuration continued; and while the patient was free from pain and could procure the necessary sleep as far as pain was concerned, he was being exhausted daily by the profuse suppuration; and his mouth was so often filled with the vile pus and other secretions that they were a source of constant irritation. During this profuse suppuration, the odor was so bad that no one would stay in the room with him. During this time he called and consulted several physicians and a dentist; two more teeth were extracted a month after the first was extracted, and many general and local remedies were used; the patient was slightly improved generally and several pieces of necrosed bone were extracted and others came away; he was treated until November last, when he was advised by a dentist in Muskegon to come to Ann Arbor, where he could have the advantages of a good hospital

The patient came to the hospital on November 6th, pale and much reduced, with a large, hard swelling on the left side of the face, over the muscles of mastication; there were several sinuses leading to necrosed bone of the lower jaw, from which considerable pus was flowing. I first saw the patient on December 3d; he was at that time, pale, weak, with cachectic appearance as if suffering from malignant disease; four sinuses lead to necrosed bone, and the swelling was so great on the left side that he could not open his mouth; it extended up to the temporal region, down on the neck, back of the ear and to the mouth, and was about as hard as a bony tumor, except in the centre where it was slightly less firm but scarcely soft enough to indicate the presence of pus. The tumor could be slightly moved independently of the jaws proving it was not a bony or cartilaginous growth attached to the jaws. One sinus opposite the left back molar lead back toward the centre of the growth for two inches, from which a small amount of pus flowed, the flow was increased by pressure on the less firm central part of the tumor, thus proving the presence of pus near the angle of the jaw. Deciding with Dr. Taft and Watling that there was pent up pus acting as an irritant, on December 4th I enlarged the sinus leading to the supposed centre of suppuration with a bistoury and groove director and put in a tent; the flow of pus was slightly increased, but drainage was incomplete as the pus was obliged to run up hill while the patient was lying down, and pressure of the tumor was so great as to close the sinus without the tent, and give the patient severe pain with the tent in the sinus, I washed out the cavity with antiseptic solution, but owing to the nature of the opening and surrounding structures, it was as far from being complete as was the drainage. On December 17th the patient was gradually growing weaker, the cachectic state more pronounced, and was also showing signs of blood poisoning; the swelling at this time was slightly less firm in the centre but growing larger. As drainage was incomplete from the internal opening, for reasons mentioned above, I considered it best to open freely externally, realizing, however, the prejudice against external openings in this region. Professor Taft agreed with me in that course, and in his presence, I made a free incision into the centre of the tumor near the angle of the jaw, close to, and parallel with the infra-maxillary nerve, considerable pus with an offensive odor, was evacuated, the parts cleansed, and warm flax seed meal poultices applied for forty-eight hours, when the acute sensitiveness was gone; the parts were then explored with a probe and a small amount of dead bone discovered near the angle of the jaw, but not yet separated; the pus cavity was found to be of irregular shape extending among the muscles of mastication. By this external opening, one of the two great indications for treating abscess was met, viz.: *free drainage*, and the other, *cleanliness*, was easily met by injecting solutions. A solution of carbolic acid and iodine was used. (A solution of 1-100 of the first, 6 parts to 1 part iodine, gradually increasing the amount of iodine.) Suppuration was free for a few days, but gradually diminished until January 15th, when a small piece of dead bone separated and was removed, after which the suppuration rapidly grew less and the sinus began to close from the bottom, stronger injections were then used. On February 13th, the sinuses were entirely closed externally and internally, the patient could open his mouth freely and masticate his food, the remaining teeth in the lower jaw had become firm in their sockets. As to general treatment, the patient, when I first saw him, being much debilitated, without appetite and bowels constipated, I gave him a non-debilitating laxative composed of aloes, nux vomica, and hyoscyamus at night, iron tonics after meals, bitter tonics before meals and to hasten the removal of the large and firm deposit of plastic matter, Iodide of Patassium 10 grs. and Bichloride of Mercury 1-16 gr. after each meal. He began to improve the next day after opening the abscess externally and has gradually improved since. A few weeks ago he was rapidly approaching death, while to-night, as you see him before you, he is a well and robust young man, a picture of perfect health. For more than a month, he has been working at manual labor about the hospital, with only a slight scar as a reminder of the serious trouble that once threatened his life.

The question naturally arises, what was the cause of this severe inflammation? We know the causes of periostitis and necrosis are in general, diseased teeth, phosphorus poisoning, syphilis, eruptive fever, excessive use of mercury, injuries, etc., but as to the causation in this case it is difficult to decide, unless it was diseased teeth, as all of the teeth extracted were found to be carious; but it is very rarely that carious teeth cause so violent a form of periostitis, for a severe periostitis which became general was the cause of the extensive suppuration and necrosis. It was not caused by mercury, for he had taken no medicine in more than seven months, and again mercurial necrosis is accompanied by greater destruction of the soft parts about the mouth. It was not phosphorus poisoning which it resembled in many of its characteristics, as the patient was never near phosphorus works, and never used *matches for tooth picks.* The patient gives no history of syphilis, after thorough questioning, and has no other signs of that much dreaded disease. If the patient had been recovering from scarlet fever, small pox, or any other eruptive disease, it would be reasonable to conclude that the eruptive fever was the cause, but no such history exists. The hard swelling, of course, was due to a deposit of plastic matter as the result of a long continued inflammation kept up by the presence of dead bone and pus. A case in some respects similar is reported in the *American Journal of Medical Science*, for 1855, page 126, by Dr. G. R. Henry, of Burlington, Iowa; the case began April 2d, 1854. The patient, a boy, of healthy parentage, always had good health till the October previous, when toothache began in the first left lower molar, and an alveo-dental abscess formed, but was not treated, the tooth was not removed, and there was a fetid discharge from about the tooth; there was a swelling over the jaw which gradually increased to a tumor resembling an encephaloid cancer, examination proving it not to be; several sinuses formed, suppuration was profuse, and the patient greatly reduced. A large part of the left side of the lower jaw became necrosed, dead bone separated, and the patient recovered. Erichsen, the famous English surgeon, in speaking of the causes of necrosis, in his excellent work on Surgery, says, in Vol. I., p. 459 (1881 edition), "I have, however, seen the disease (speaking of necrosis of the lower jaw) occur in otherwise healthy persons without any assignable cause; in this way, I have seen the whole of the alveolar process of the upper jaw exfoliate in a young lady otherwise perfectly healthy; and I have several times had occasion to remove large portions of the lower jaw—in one case, more than half of the bone for necrosis that could not be referred to any assignable cause. The disease begins with a deeply seated pain, resembling inveterate toothache, which nothing will allay; the gums become swollen; teeth loosen and drop out; pus wells up from the aveoli; abscesses inside of the mouth and at the angle of the jaw form, and fistulous openings through which dead bone can be felt with a probe. The patient's health suffers greatly; more than in necrosis generally, doubtless in consequence of the patient swallowing so much of the pus from the dead bone."

This positive statement, coming from so eminent a surgeon as Dr. Erichsen assures us that necrosis of the lower jaw may come from causes that cannot be discovered. This is of importance to members of the dental profession, who are often unjustly accused of producing diseases with which they had no connection whatever.

It might not be out of place here to say a few words with reference to treatment of necrosis of the lower jaw. The treatment naturally divides itself into preventative and curative.

In all such cases, the question presents itself: Could this disease have been so modified that these serious destructive results could have been prevented? It should always be remembered that in the early stages, where there is heat, swelling, redness, a deep distensile pain in the region of lower jaw, and a general feverish state, that we have an active inflammation of a rapidly destructive nature to deal with; a periostitis generally—sometimes, more rarely, an osteïtis, and sometimes both, and it should be combatted by active antiphlogistic remedies early and *persistently*, for, as Dr. Heath, in his standard work on "Diseases of the Jaws," says: "Such an inflammation may make necrosis of the lower jaw inevitable within a short time, and sometimes within a few hours."

The plan of treatment that I would suggest after consulting many authorities is as follows: If seen in the early active stage, give a good brisk cathartic, say 6 to 10 grs. of calomel or blue mass, followed in from four to eight hours by a large dose of saline cathartic, well diluted; follow this by 10 to 15 grs. of Dover's powder or equivalent of some other opiate, to allay all pain, and repeat the dose as often as needed. If the pulse be hard, active, and the skin hot and dry, give drop doses of tinc. aconite every half hour in water until the skin is moist and the action of the heart is modified. By the action of these remedies, the blood is diverted from the part, the pain checked, the irritation quieted, the force of the heart modified, and the general temperature lowered; in other words, the part put at rest, which is the great essential in inflammation wherever located. It is of the utmost importance to keep the bowels active whenever there is inflammation about the head or face, for by calling the blood, by brisk cathartics to the 25 or 30 ft. of intestines, it is diverted from the head. Scarify the gums freely by free incisions down to the bone for two reasons: 1st, local blood-letting relieves the blood pressure, and 2nd, it gives free vent to any pus that may collect under the periosteum, thus preventing extensive suppuration of the periosteum and extensive necrosis in many cases; the gums may be painted with tinc. iodine, hot fomentations applied over the swollen parts, and such remedies. The treatment thus far is to combat periostitis and osteitis, and to prevent necrosis, caries, or abscess, which are the results of continued inflammation.

Whenever necrosis of the lower jaw is discovered, the best method to pursue, according to most British and American surgeons, is to meet indications generally and locally until the necrosed bone is separated from the living bone and then assist nature in getting rid of the irritant by enlarging the opening and removing the sequestrum or sequestra, as the case may be, avoiding injuring the large blood vessels and nerves in the removal.

The indications to be met generally are with supporting remedies, as iron and bitter tonics; and in specific cases, alteratives; without alteratives in specific cases, the patient cannot be cured, but with them, syphilis yields promptly.

The suppuration being profuse, the patient, in many cases, is rapidly debilitated, and nourishing food, tonics, and sometimes stimulants, are plainly indicated to assist nature by supporting her. In these cases the supporting powers of quinine cannot be over-estimated. Locally, the sinuses should be cleaned by stimulating, alterative, or astringent injections as indicated, as solutions of iodine, listerine, peroxide of hydrogen, carbolic acid, etc., and the mouth should be cleaned often with solutions of permanganate of potash, listerine, etc., etc. As a rule, we cannot improve upon Nature's methods, and our duties as physicians and dentists are to discover Nature's methods and to imitate her and assist her by removing all obstacles to her processes.

